# Neonatal Thyroid-Stimulating Hormone Concentrations in Belgium: A Useful Indicator for Detecting Mild Iodine Deficiency?

**DOI:** 10.1371/journal.pone.0047770

**Published:** 2012-10-24

**Authors:** Stefanie Vandevijvere, Wim Coucke, Jean Vanderpas, Caroline Trumpff, Maarten Fauvart, Ann Meulemans, Sandrine Marie, Marie-Françoise Vincent, Roland Schoos, François Boemer, Timothy Vanwynsberghe, Eddy Philips, François Eyskens, Brigitte Wuyts, Valbona Selimaj, Bart Van Overmeire, Christine Kirkpatrick, Herman Van Oyen, Rodrigo Moreno-Reyes

**Affiliations:** 1 Scientific Institute of Public Health, Brussels, Belgium; 2 Université Libre de Bruxelles, Laboratoire de Pédiatrie, Brussels, Belgium; 3 Université Catholique de Louvain, Louvain-La-Neuve, Belgium; 4 Biochemical Genetics Laboratory, CHU Sart-Tilman, Liège, Belgium; 5 AZ Sint-Jan Brugge, Brugge, Belgium; 6 Provinciaal Centrum voor de Opsporing van Metabole Aandoeningen, Antwerpen, Belgium; 7 Laboratory of Metabolic Disorders, UZ Gent, Gent, Belgium; 8 Université Libre de Bruxelles, Hôpital Erasme, Bruxelles, Belgium; Aga Khan University, Pakistan

## Abstract

It has been proposed that neonatal thyroid-stimulating hormone (TSH) concentrations are a good indicator of iodine deficiency in the population. A frequency of neonatal TSH concentrations above 5 mU/L below 3% has been proposed as the threshold indicating iodine sufficiency. The objective of the present study was to evaluate feasibility and usefulness of nation-wide neonatal TSH concentration screening results to assess iodine status in Belgium. All newborns born in Belgium during the period 2009–2011 (n = 377713) were included in the study, except those suffering from congenital hypothyroidism and premature neonates. The frequency of neonatal TSH concentrations above 5 mU/L from 2009 to 2011 in Belgium fluctuated between 2.6 and 3.3% in the centres using the same TSH assay. There was a significant inverse association between neonatal TSH level and birth weight. The longer the duration between birth and screening, the lower the TSH level. Neonatal TSH levels were significantly lower in winter than in spring or autumn and significantly lower in spring and summer than in autumn while significantly higher in spring compared to summer. In conclusion, despite that pregnant women in Belgium are mildly iodine deficient, the frequency of neonatal TSH concentrations above 5 mU/L was very low, suggesting that the neonatal TSH threshold proposed for detecting iodine deficiency needs to be re-evaluated. Although neonatal TSH is useful to detect severe iodine deficiency, it should not be recommended presently for the evaluation of iodine status in mildly iodine deficient regions.

## Introduction

Along with urinary iodine concentrations (UIC), it has been proposed that neonatal thyroid-stimulating hormone (TSH) concentrations are a good indicator for the prevalence of iodine deficiency disorders (IDD) in the population [1]–[3]. Elevated neonatal serum TSH concentration may indicate insufficient supply of thyroid hormones to the developing foetal brain, and is therefore the only measure that allows prediction of brain damage due to iodine deficiency [2]. The neonatal thyroid has a low iodine content compared to the adult thyroid, and hence neonatal iodine turnover is much higher. In case of iodine deficiency, this turnover is even higher, stimulating thereby TSH secretion. Consequently it has been assumed that the neonatal thyroid is extremely sensitive to iodine deficiency.

The World Health Organization (WHO) has proposed to use the results of screening programs for congenital hypothyroidism in neonates as an additional index for the evaluation of iodine status of the population. A frequency of neonatal TSH concentrations above 5 mU/L below 3% was proposed as indicating iodine sufficiency. In mild iodine deficiency (MID) the frequency may be 3–19.9% and the frequencies of 20–39.9% and above 40% may be found in moderate and severe iodine deficiency respectively [2]; [4]. Although TSH levels in neonates have already been used previously for assessing the prevalence of iodine deficiency [5]–[8], the cut-off of 5 mU/L, as set by the WHO, has been criticized [9]–[11].

Some studies suggest that MID during pregnancy may impair neurodevelopment in the offspring [12] and prevent them from reaching their full intellectual potential. Correction of mild-to-moderate iodine deficiency in primary school children was found to improve cognitive and motor function [13].

Despite a worldwide successful implementation of iodine supplementation programs over the last decades, iodine deficiency remains a public health problem in Europe [14]. Since 2003, the number of European countries in which iodine deficiency remains a public health problem decreased from 23 to 14 [15], but it is a matter of concern that iodine deficiency has re-emerged in countries thought to have sufficient iodine intake, such as the UK [16]. Several surveys in the past indicated that Belgium is affected by MID, and that this represents a substantial economic burden to the Belgian health care system [17]–[19]. A recent national survey on iodine status in Belgian school-aged children showed a median UIC of 113.1 µg/L while 84.4 µg/L among their mothers, indicating iodine sufficiency among children and MID among their mothers [20]. The median UIC in Belgian pregnant women (124.1 µg/L) also indicated MID, as it was below 150 µg/L. Of all pregnant women included, 59.3% had a UIC below 150 µg/L and 37.8% had a UIC below 100 µg/L [21].

The objective of the present study was to evaluate feasibility and usefulness of nation-wide neonatal TSH concentration screening results to assess iodine status in Belgium. In order to compare TSH concentrations among different laboratories, a ring test was organized among the different centres in Belgium screening for congenital hypothyroidism. In addition, factors influencing neonatal TSH concentrations in Belgium were investigated.

## Methods

### Ethics Statement

For the purpose of this study, already collected data were used. Due to the huge sample size no informed consent was obtained from the parents of the neonates in order to seek approval for the use of the data for the purpose of this study. This study was approved by the Privacy Commission in Belgium. The Privacy Commission waived the need for written informed consent form in order to use the data. The ring test was approved by the medical ethical committee of the Erasme hospital and mothers of neonates provided oral and written informed consent for participation of their neonate in the ring test.

### Data Collection

In Belgium there are 6 screening centers for congenital hypothyroidism spread all over the country. Since 1976 all neonates born in Belgium are screened for congenital hypothyroidism on day 3 to 5 after birth. Absorbing cards are distributed to the obstetric clinics by the screening centres. Venous blood is collected directly on the cards by the nurses via a heel stick. All cards contain a unique identification code for each child. On each card 4 circles are drawn with a diameter of 10 mm to collect the blood spots. The blood is distributed equally over the circles and the circles are filled completely. The remaining 3 circles are used for the screening of other metabolic diseases. Following parameters are collected on the blood card for each newborn: identification of the clinic, unique number of the child, hospital of birth or general practitioner/midwife if born out of hospital, sex of the child, birth date of the child, type of feeding (breastfeeding), medications, duration of pregnancy and birth weight.

Blood cards are dried at room temperature and transported within 24 hours to the responsible screening centre for the analysis.

### Analysis of Dried Blood Samples

For the quantitative determination of TSH on filter paper, a time-resolved fluorimmoassay (Autodelfia or Delfia method) was used by 4 out of 6 centres. This TSH assay is a solid phase, two-site fluorimmunometric assay based on the direct sandwich technique in which two monoclonal antibodies are directed against two separate antigenic determinants on the TSH molecule [22]. The analytical sensitivity of the assay is 2 µU/mL.

An in-house enzyme-linked immunosorbent assay (ELISA) was used by 1 centre to quantify TSH in blood specimens dried on filter paper. A 3 mm spot of calibrator or sample or internal control was incubated during one night at 4°C to be fixed to a capture monoclonal anti TSH antibody coated on the wells of microplates. After washing, a solution of a second monoclonal antibody chemically bound to horseradish peroxydase was added and incubated for one hour at room temperature. After a new washing step, a blue colour was developed for 10 minutes by adding the substrate (H_2_O_2_) and the chromogen (TMB) solution. After stopping with an acidic solution, the colour intensity was measured by a spectrophotometer microplater reader at 450 nm. The NIBSC 81/585 standard for TSH was used to prepare the dried blood calibrators [23]. The analytical sensitivity of the method is 3.9 mU/L.

A commercial ELISA method, the sandwich enzyme-immunoassay (EIA), the Quantase TM Neonatal Screening Assay provided by Biorad, was used by 1 out of 6 screening centres. The sample was incubated with a peroxidase-labelled anti-TSH monoclonal antibody in a micro well coated with another anti-TSH monoclonal antibody. After incubation, the wells were washed free of unbound labeled antibody. TSH in the sample was determined by the reaction of the bound conjugate with substrate (TMB) producing a coloured product. The concentration of TSH in the sample was proportional to the colour measured at 450 nm in a plate reader. The analytical sensitivity of the method is 1.96 µU/mL.

In case of an increased TSH level (>15 mU/L or >20 mU/L, depending on the analysis methodology used), an extra investigation is performed at the hospital, for eventual detection of permanent primary hypothyroidism.

### Ring Test

Lack of method standardization is a major drawback in comparing data from different centres. A ring test is useful to be able to facilitate intercentre comparisons and to calibrate laboratories to each other to compare TSH level between centres and/or analysis methodologies. This ring study was approved by the medical ethical committee of the Erasme hospital in Brussels. In this study blood from 100 neonates was collected at the maternity of the Erasme hospital in Brussels using a heparanized microtube. A blood spot of 50 µl was placed on the special filter paper (S&S 903). After drying, these filter papers were sent to the six screening centres in order to determine TSH concentrations.

### Statistical Analysis

A method and laboratory comparison using the results from the samples was performed using the Deming regression model [24]. Whereas the ordinary linear regression method assumes that only the Y measurements are associated with random measurement errors, the Deming method takes measurement errors for both Y and X measurements into account. For centre 5, using the ELISA in-house method and centre 6, using the commercial ELISA method, regression equations were determined to be able to convert the TSH values from their method to the (Auto)delfia method used by centre 1, 2, 3 and 4. The standard errors and confidence intervals of the intercept and slope were estimated using bootstrapping.

Because of the high variability between different analysis methods, it was decided to use 6 different scenarios in order to present neonatal TSH concentrations for Belgium over the different years. In scenario 1 the regression equations used the point estimators for both slope and intercept. In scenario 2 the lower limit of the confidence interval (CI) was used for both intercept and slope while in scenario 3 the higher limits of intercept and slope were used. In scenario 4 the lower limit of CI for intercept and the higher limit of CI for slope were used and in scenario 5 the other way around. In scenario 6 only the neonatal TSH values from the first four centres using the same analysis method were used to compare TSH over the different years.

Since 2009 TSH levels of all neonates in Belgium are electronically available by all 6 centres and collected in a central database on a yearly basis. Neonates were excluded from this study if they were affected by primary neonatal hypothyroidism and if they were premature. In the first case all neonates with a TSH level higher than 15 mU/L were excluded. Prematurity was defined as a birth weight lower than 2500 g and/or pregnancy duration lower than 37 weeks.

TSH values were log transformed after adding 1. Differences in neonatal TSH levels in Belgium among the different years (2009, 2010, and 2011) were explored using analysis of variance (ANOVA). Multiple pair wise comparisons were made with the Tukey test.

Finally, to evaluate the effect of the factors birth weight, pregnancy duration, duration between birth and screening date, duration between birth and analysis date, year, sex and season on neonatal TSH level, linear mixed models were used with screening centre included as random effect.

## Results

### Ring Test

**Figure 1:**
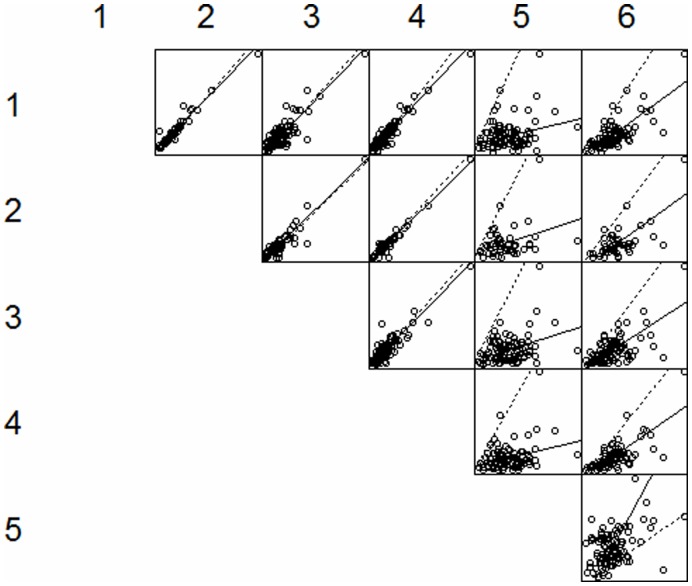
Results of Deming regression among the 6 neonatal screening centres for congenital hypothyroidism in Belgium. (Full line = Deming regression line; dotted line = 45° line). Centre 1: Brussels; Centre 2: Gent; Centre 3: Brugge; Centre 4 : Antwerpen; Centre 5 : Liège; Centre 6 : Louvain en Woluwé.

**Figure 2:**
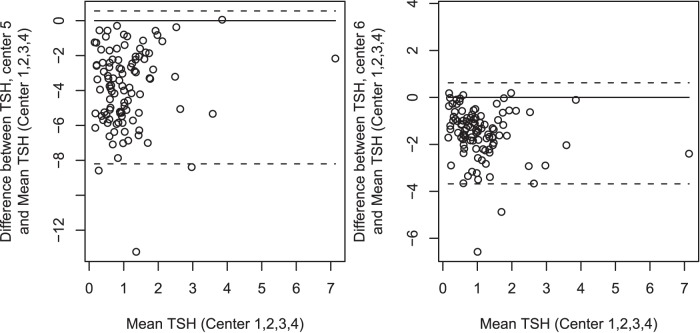
Bland Altman plot of comparison of Method 1 (Autodelphia) used by centre 1,2,3,4 versus Method 2 (ELISA commercial Biorad) used by centre 5. Mean difference: −3.854 mU/L (CI −4.291 to −3.417 mU/L) (left) and method 1 (Autodelphia) used by centre 1,2,3,4 versus method 3 (ELISA) used by centre 6; mean difference: −1.470 mU/L (CI −1.692 to −1.249 mU/L) (right).

Results from the Deming regression comparing the different centres can be found in Figure 1. There is a very good agreement between the 4 centres using the (Auto)delfia method. There are only significant differences between centres 1,2,3,4 and both centre 5 and 6. Variability is high comparing centre 5 and 6 to the other 4 centres (Figure 2). The regression equations to convert TSH values of centre 5 and 6 to the Autodelfia method are shown in Table 1. The 95% confidence interval for the intercept shows that the intercept is not significantly different from 0 in both cases, which means that both the analysis method of centre 5 and centre 6 do not significantly differ with a constant amount from the Autodelfia method. The 95% confidence interval for the slope shows that in both cases the slope is significantly different from 1 and there is a proportional difference between the both the method of centre 5 and 6 and the Autodelfia method.

**Table 1:** Regression equations and 95% CI to convert TSH levels of centre 5 and 6 to Autodelphia method.

centre	intercept	intercept, CI(low)	intercept, CI(high)	slope	slope, CI(low)	slope, CI(high)
5	0,44	−0.365	0.978	0.135	0.013	0.319
6	−0.201	−0.629	0.334	0.496	0.257	0.68

Centre 5: Liège; Centre 6: Louvain en Woluwé. CI confidence interval.

### TSH Concentrations in Neonates in Belgium

In Table 2 median and mean TSH values over the three different years and using the 6 different scenarios are shown. For all scenarios all pair wise comparisons between the different years were significantly different.

**Table 2:** Distribution of TSH values (between 0 and 15 mU/L) over the three different years by the six different scenarios.

	2009 (n = 126715)						2010 (n = 131548)						2011 (n = 119450)							p
	median	mean	stdev	P95	% >5 mU/L	% >2 mU/L	median	mean	stdev	P95	% >5 mU/L	% >2 mU/L	median	mean	stdev	P95	% >5 mU/L		% >2 mU/L	
TSH1	1.2	1.5	1.2	3.8	2.0	23.4	1.3	1.7	1.4	4.4	3.5	29.7	1.3	1.6	1.3	4.0	2.4		27.6	***
TSH2	1.0	1.3	1.3	3.7	1.9	21,0	1.1	1.4	1.4	3.9	2.2	24.4	1.0	1.3	1.4	3.9	2.3		22.9	***
TSH3	1.6	1.9	1.3	4.2	2.7	36.0	1.7	2.1	1.7	5.4	6.1	39.4	1.7	2.0	1.4	4.6	3.7		40.6	***
TSH4	1.3	1.5	1.3	3.9	2.2	25.6	1.4	1.8	1.6	4.9	4.7	31.3	1.4	1.7	1.4	4.2	2.9		30.2	***
TSH5	1.1	1.5	1.2	3.7	1.9	21.5	1.3	1.7	1.3	3.9	2.3	26.9	1.2	1.6	1.2	3.9	2.3		24.1	***
	**2009 (n = 91107)**						**2010 (n = 92961)**						**2011 (n = 81511)**							**p**
TSH6	1.4	1.7	1.32	4.1	2.6	29.1	1.5	1.8	1.4	4.3	3.0	32.1	1.5	1.8	1.4	4.4	3.3	33.3		***

*** P<0.05 for all Tukey tests comparing differences in TSH levels over the years 2009, 2010, 2011. TSH1: TSH values of centre 5 and 6 converted to Autodelphia method using point estimators for intercept and slope. TSH2: TSH values of centre 5 and 6 converted to Audodelphia method using lower limit of CI for intercept and slope. TSH3: TSH values of centre 5 and 6 converted to Autodelphia method using higher limit of CI for intercept and slope. TSH4: TSH values of centre 5 and 6 converted to Autodelphia method using lower limit of CI for intercept and higher limit of CI for slope. TSH5: TSH values of centre 5 and 6 converted to Autodelphia method using lower limit of CI for slope and higher limit of CI for intercept. TSH6: Only TSH values of 4 centres using Autodelphia method.

For all scenarios TSH levels were highest in 2010 and decreased again in 2011. Taking into account only the four centres using the same method, TSH level slightly increased from 2009 to 2011. According to the WHO criteria, the iodine status of Belgian neonates is optimal or borderline sufficient as the percentage of neonates with a TSH level higher than 5 mU/L is either lower than 3% or around 3% for the different scenarios, except in 2010 for the scenarios using the upper limit of CI for the slope and/or intercept.

### Factors Affecting Neonatal TSH Concentrations in Newborns

Extra information on birth weight, pregnancy duration, sex of the neonate, season and duration between birth and screening, was only available for 3 out of 6 centres (n = 78004). There was a significant inverse association between neonatal TSH level and birth weight while there was a significant positive association between neonatal TSH level and pregnancy duration. The longer the duration between birth and screening or birth and analysis, the lower the TSH level (data not shown). In order to explore the differences in TSH concentration over the different years and seasons a mixed model was used. Differences of least square means between years and seasons can be found in Table 3. Differences in neonatal TSH concentrations between 2010 and 2011 were not significantly different but neonatal TSH levels were significantly lower in 2009 than in 2010 and 2011. Neonatal TSH levels were significantly lower in winter than in spring or autumn and lower in spring and summer than in autumn while significantly higher in spring compared to summer. Results were the same in all 5 scenarios. In the scenario only taking into account the 4 centres with the same method, TSH was significantly lower in 2010 and 2009 compared to 2011 but additional factors could not be taken into account in this model.

**Table 3:** Least square means of difference in TSH among years and seasons, linear mixed model regression.

Effect		Estimate*	p
Year	2009–2010	−0.035	<0.001
Year	2009–2011	−0.044	<0.001
Year	2010–2011	−0.009	0.057
season	winter-spring	−0.015	<0.001
season	winter-summer	−0.006	0.110
season	winter-autumn	−0.026	<0.001
season	spring-summer	0.009	0.010
season	spring-autumn	−0.011	0.004
season	summer-autumn	−0.020	<0.001

* transformed values.

## Discussion

The frequency of neonatal TSH concentrations above 5 mU/L in Belgium was unexpectedly low considering that pregnant women in Belgium are mildly iodine deficient. This percentage fluctuated between 2.6 and 3.3% from 2009 to 2011 in the centres using the same TSH assay.

The WHO proposed that when a sensitive assay is used on samples collected 3–4 days after birth a frequency of TSH concentrations >5 mU/L less than 3% indicates iodine sufficiency in the population [4]. However, although several studies attempted to apply neonatal TSH values in determining population iodine status and some have proven to be successful [5]; [6]; [8]; [25], some have provided conflicting or uncertain data [10]; [11].

The utilisation of neonatal TSH is an attractive method because it is assumed that the thyroid of the newborn is very sensitive to iodine status and even MID during pregnancy will cause an increase in neonatal TSH secretion. In addition in countries where such screening programme exists, the utilisation of neonatal TSH concentrations does not imply an extra cost. Unfortunately in most of the regions where iodine deficiency is severe such screening programs are usually lacking. In Belgium however the situation is complicated because there are 6 screening centres using 3 different assay methods. Despite this heterogeneity and after applying correction factors, all the centres showed concordant values indicating a low frequency of TSH >5 mU/L.

In case of severe iodine deficiency neonatal TSH concentrations are consistently high [3] and most of the conflicting results on neonatal TSH for the evaluation of iodine status concern mildly iodine deficient regions. A TSH frequency >5 mU/L below 3% has been reported in several mildly iodine deficient regions suggesting that neonatal TSH may not be sensitive enough to evaluate iodine status when iodine deficiency is mild. A study from Australia using a sensitive TSH assay found that only 2.2% of neonates had a TSH value >5% despite a median UIC of 85 µg/l among pregnant women [26]. In Belgium iodine status is characterized by iodine sufficiency in school-aged children and MID in pregnant women and in women of child-bearing age [20]; [21]. Despite that pregnant women are mildly iodine deficient the frequency of TSH values >5 mU/L in Belgium was low, considering the results from the centres using the same TSH assay, corroborating previous studies from mildly iodine deficient regions [26]. Interestingly, a clinical trial performed in Belgium on iodine supplementation in pregnant women did show differences in neonatal thyroglobulin concentrations but not in TSH levels of neonates born from women who received iodine supplements compared to those who did not [27]. The change in median UIC after iodine supplementation derived from the data published was probably lower than 100 µg/L. This variation of iodine intake reflected by an increase of the median UIC lower than 100 µg/L may be too small to induce an increase in neonatal TSH but may be capable of triggering an increase of neonatal thyroglobulin concentrations.

In Switzerland the frequency of neonatal TSH >5 mU/L decreased significantly from 2.9% to 1.7% with the increase in salt iodine concentrations [28]. However this modification of TSH frequency was associated with an increase in the median UIC in pregnant women greater than 100 µg/L; from 138 µg/l to 249 µg/L. Altogether these results suggest that a low frequency of TSH >5 mU/L cannot exclude MID during pregnancy or cannot alone detect a small variation of iodine intake in the population. It is possible that instead of applying a single cut-off of blood TSH level, the trend of TSH concentrations may provide an early indication of impending iodine deficiency as suggested in a study from Ireland [9].

Several factors, other than iodine intake influence neonatal TSH concentration and may explain to some extent the conflicting results when applied for the monitoring of iodine status. One of the most relevant factors is related to the considerable variations of TSH values obtained with the different available TSH assays as shown in the present study. However in earlier publications determining the appropriate neonatal TSH cut-off to detect iodine deficiency this issue was not addressed [3] and it is possible that some of the conflicting results may arise from a cut-off derived from studies using different TSH assays. In addition neonatal TSH is also affected by the mode of delivery [29], smoking behaviour of the mother [30]; [31], use of iodine-containing antiseptics during delivery [32] and multiple pregnancies [33].

In the present study, an inverse association was found between neonatal TSH levels and birth weight and an inverse association was found with duration between birth and screening, as shown previously [34]. In a single centre in Northern England males seemed to have higher TSH levels than females [35]. In Belgium there was no difference in TSH levels between boys and girls. In addition the frequency of neonatal transient hyperthyrotropinemia appears significantly less in autumn than in spring, [36] such as found also in the present study.

Although the use of neonatal TSH concentrations is attractive for monitoring iodine status, many issues need to be addressed before it can be recommended for this purpose, particularly in mildly iodine deficient countries. The sensitivity of neonatal TSH concentration in conditions of MID needs to be reassessed in view of the conflicting data, suggesting that neonatal TSH may not be as sensitive as previously thought. The hypersensitivity of the neonatal thyroid to small variations of iodine intake also needs to be re-evaluated and compared to neonatal thyroglobulin concentrations that may be more sensitive to smaller variations of iodine intake during pregnancy. Finally the use of standard protocols for sample blood collection and TSH assays is mandatory to be able to compare results from different regions and to define appropriate cut-offs.

In conclusion, despite that pregnant women in Belgium are mildly iodine deficient, the frequency of neonatal TSH values above 5 mU/L was unexpectedly low suggesting that the TSH threshold proposed for detecting iodine deficiency needs to be re-evaluated. Although neonatal TSH is useful to detect severe iodine deficiency it should not be recommended presently for the evaluation of iodine status in mildly iodine deficient regions.
